# Peer Review of “Monte Carlo Dose Estimation of Absorbed Dose to the Hematopoietic Stem Cell Layer of the Bone Marrow Assuming Nonuniform Distribution Around the Vascular Endothelium of the Bone Marrow: Simulation and Analysis Study”

**DOI:** 10.2196/77776

**Published:** 2025-07-16

**Authors:** Maha Gasmi

**Affiliations:** 1 Manouba University, Manouba, Tunisia

**Keywords:** stem cells, radiation, bone marrow, nuclides, noble gases

*This is the peer-review report for “Monte Carlo Dose Estimation of Absorbed Dose to the Hematopoietic Stem Cell Layer of the Bone Marrow Assuming Nonuniform Distribution Around the Vascular Endothelium of the Bone Marrow: Simulation and Analysis Study.”*

## Round 1 Review

### Abstract Section

1. The manuscript’s [1] abstract begins with a statement about hematopoietic stem cells’ proximity to sinusoidal capillaries but does not clarify why this spatial distribution is relevant for radiation dosimetry until later in the text. A clearer explanation linking the hematopoietic stem cell location with the dosimetric model limitations would better engage readers unfamiliar with the topic.

2. Some sentences are overly complex, especially in the Introduction and Conclusion. Simplifying the language or splitting ideas across multiple sentences could improve readability.

3. The abstract lacks methodological detail regarding how the model calculations were performed. Including brief specifics about the model’s approach, particularly the role of computed tomography imaging if applicable, would improve transparency and give context to the reported findings.

4. The results comparing the absorbed doses for α and β nuclides are presented with limited interpretation. The abstract states that doses for β nuclides were similar to International Commission on Radiological Protection estimates, while those for α nuclides were much lower, yet there is no explanation for the potential reasons behind these differences. Offering a brief discussion or hypothesis, even speculative, would enrich the reader’s understanding.

### Introduction Section

5. The Introduction could benefit from a clearer structure. Currently, it presents information about various models and dosimetric approaches in a somewhat fragmented manner.

6. Certain technical terms such as “surrogate target,” “trabecular bone surface,” “endosteum,” and “standard absorbed fraction” may benefit from concise explanations or definitions. For instance, briefly defining “surrogate target” would help those unfamiliar with dosimetry or radiobiology terminology.

### Method Section

7. The study uses an intricate geometric model based on JM-103 data, Particle and Heavy Ion Transport System software, and Japan Atomic Energy Agency guidelines to simulate the cervical vertebrae trabecular bone. This choice is reasonable given the need for anatomical detail in dosimetry but may limit generalizability since the cervical vertebrae structure might not fully represent other bone marrow sites.

The description could benefit from clarifying why the JM-103 model was chosen over other models or datasets, particularly those that could include bone tissues beyond the cervical vertebrae.

### Discussion Section

8. Despite noting the need for micro–computed tomgraphy–based models, the authors do not discuss how current limitations might impact dose estimation accuracy, especially for complex geometries in the trabecular bone. A clearer explanation of how simplified geometric assumptions may influence absorbed dose calculations would provide a balanced view of the model’s limitations.
